# The Role of Natural Killer Cells in the Immune Response in Kidney Transplantation

**DOI:** 10.3389/fimmu.2020.01454

**Published:** 2020-07-23

**Authors:** Paola Pontrelli, Federica Rascio, Giuseppe Castellano, Giuseppe Grandaliano, Loreto Gesualdo, Giovanni Stallone

**Affiliations:** ^1^Nephrology, Dialysis and Transplantation Unit, Department of Emergency and Organ Transplantation, University of Bari Aldo Moro, Bari, Italy; ^2^Nephrology, Dialysis and Transplantation Unit, Department of Medical and Surgical Sciences, University of Foggia, Foggia, Italy; ^3^Nephrology Unit, Department of Translational Medicine and Surgery, Università Cattolica del Sacro Cuore, Rome, Italy; ^4^Fondazione Policlinico Universitario “A. Gemelli” IRCCS, Rome, Italy

**Keywords:** natural killer cells, innate and adaptive immune response, kidney graft rejection, tolerance, immunosuppression

## Abstract

Natural killer cells (NK) represent a population of lymphocytes involved in innate immune response. In addition to their role in anti-viral and anti-tumor defense, they also regulate several aspects of the allo-immune response in kidney transplant recipients. Growing evidence suggests a key role of NK cells in the pathogenesis of immune-mediated graft damage in kidney transplantation. Specific NK cell subsets are associated with operational tolerance in kidney transplant patients. On the other side, allo-reactive NK cells are associated with chronic antibody-mediated rejection and graft loss. Moreover, NK cells can prime the adaptive immune system and promote the migration of other immune cells, such as dendritic cells, into the graft leading to an increased allo-immune response and, eventually, to chronic graft rejection. Finally, activated NK cells can infiltrate the transplanted kidney and cause a direct graft damage. Interestingly, immunosuppression can influence NK cell numbers and function, thus causing an increased risk of post-transplant neoplasia or infection. In this review, we will describe how these cells can influence the innate and the adaptive immune response in kidney transplantation and how immunosuppression can modulate NK behavior.

## Phenotype, Maturation, Cytotoxic Activity, and Distribution of Natural Killer (NK) Cells

Natural killer (NK) cells are effector lymphocytes deriving from common lymphoid progenitors and represent 5–10% of circulating lymphocytes. NK cells are natural cytotoxic cells, but, unlike cytotoxic T lymphocytes, they do not require antigen exposure to mediate their effect (1). NK cells represent one of the main cellular components of innate immunity along with mast cells, eosinophils, basophils, macrophages, neutrophils, and dendritic cells. They mediate immune responses against intracellular pathogens representing key mediators of the anti-viral and anti-neoplastic defense, but they also play a key role, through the production and release of several cytokines, in many inflammatory diseases, including acute and chronic kidney diseases (2–4). Interestingly, the role of this lymphocyte subset in the progression of kidney injury is starting to be uncovered (5).

The NK cells accomplish their cytolytic effector activity through two main mechanisms of action (6):

1. **Direct lysis**. The recognition of HLA class I molecules by inhibitory receptors (KIRs: Killer cell immunoglobulin-like receptors) on NK cells inhibits their cytotoxic activity and maintains the recognition of self. In the case of “missing self” instead, the absence of class I HLA molecules on target cells (e.g., cancer cells) prevents inhibitory signals from switching off the cytotoxicity of NK cells.
2. **Antibody-dependent cellular cytotoxicity (ADCC)**. The interaction between the Fc receptor FcγRIII (CD16) expressed on NK cells and the Fc fragment of an antibody recognizing foreign antigens on target cells (e.g., infected cells) induces the lysis of these cells.

In both cases, the lytic function of NK cells depends upon cytolytic molecules, mainly granzyme and perforin, and their activation leads to the production of several inflammatory cytokines (7). Granzyme and perforin are included into cytoplasmic lytic granules, characterized by several lysosomal-associated membrane glycoproteins (LAMPs) into the lipid bilayer. These proteins appear on cell surface after cytotoxic granules exocytosis (8). Among the different LAMPs, CD107a/LAMP-1 has been widely used as a functional marker to identify NK cell activity, since its expression is significantly higher on the surface of NK cells after MHC stimulation and correlates with both cytokine secretion and NK cell-mediated lysis of target cells (9, 10). Interestingly, Conehn et al. demonstrated that CD107a/LAMP-1 protects NK cells from self-destruction upon target cell killing, since CD107a/LAMP-1 deficiency, both in human and in mice NK cells, increased NK cell apoptosis after degranulation (11).

The interaction of NK cells with the target cell can occur through distinct inhibitory or stimulatory receptors and, therefore, defines the fate of the target cell (12). Normal cells are protected from NK cell killing since stimulatory receptors signals are balanced by inhibitory receptors signals coming from the interaction with the self-molecules of the MHC class I complex. Neoplastic transformation or cellular infection can induce the expression of stimulatory ligands that overcome the inhibition induced by inhibitory receptors. In this case, an induced-cell recognition occurs (12). In many contexts both missing-self and induced-self recognition are likely to operate simultaneously to provide to NK cells the maximum ability to discriminate normal cells from transformed or infected ones.

NK cells can express on their surface different receptors able to recognize several polymorphic variants of MHC I molecules. Indeed, the human NK cell receptor repertoire is highly complex in each individual (13). In addition, NK cells express specific stimulatory and inhibitory receptors for various other ligands present on the surface of the target cells and the balance of inhibitory and stimulatory signals received by a NK cell determines the outcome of its interactions with target cells (14). These signals involve immunoreceptor tyrosine-based activation motif (ITAM)-bearing molecules and inhibitory receptors, other stimulatory receptors and adhesion molecules, such as KIR, immunoglobulin-like transcript (LIR), leukocyte-associated immunoglobulin-like receptor (LAIR), vascular cell adhesion molecule-1 (VCAM-1), intercellular adhesion molecule (ICAM) (14). Thus, the activation program of NK cells derives from the integration of activator and inhibitor signals, which varies according to the nature of the interacting cells. In addition, NK cells also express cytokines and chemokines receptors that are crucial for the regulation of NK cell functions and Toll like receptors that mediate the production of IFN-g and increase cytotoxicity (15).

NK cell maturation can occur in the medulla of secondary lymphoid organs, lymph nodes, tonsils, and spleen (16). The hematopoietic stem cells Lin–CD34+ can differentiate into multipotent lymphoid progenitors (LMPP) CD45RA+, expressing the stem marker CD34. The expression by multipotent lymphoid progenitors of CD38, CD7, CD10, and the cytokine receptor CD127 (IL-7 receptor-alpha) drives the transition to common lymphoid progenitors that, in turn, may generate progenitors of T cells, B cells, NK cells, and other innate lymphoid cells (ILCs). An ILC restricted progenitor can origin two main ILC lineages, Killer ILC and helper-like ILC (17). Helper-like ILCs express IL-7R-alpha, require GATA-3 for differentiation, and are composed of various cytokine-producing ILC subsets: ILC1, ILC2, and ILC3. ILC1 expresses and requires the transcription factor T-bet for lineage specification and produces large amounts of IFN-g (18). ILC2 produces type 2 cytokines and amphiregulin (19), thus driving type 2 adaptive immune responses through activation of Th2 cells, ILC3 express type 17 cytokines such as IL-22 and IL-17A (20). Similarities between helper-like ILC and T cell subsets led us to propose ILC as the innate counterparts of T cell subsets (21). The expression of CD122 (IL-2 receptor beta) marks the irreversible commitment of common lymphoid progenitors to give rise to conventional NK cells. Conventional NK cells and helper-like ILC1 can be distinguished by the expression of some transcription factors such as the T-box protein in T cells (Tbet) and Eomesodermin (Eomes) since mature NK are Tbet+ Eomes+ while ILC1 are Tbet+ Eomes-; however, the distinction between NK cells and other ILC populations also concerns their cytotoxic properties and other molecules (such as CD200r1, Eomes, CD49b) expressed in different organs and different activation states (22) that can drive different response during homeostasis and viral-induced inflammation (23). Finally, the appearance of the adhesion molecule CD56 (NCAM: neural cell adhesion molecule) designates the final transition of immature NK cells toward a mature phenotype (16).

The NK cell maturation gives rise to a small population of CD56^bright^ (5%) cells, expressing high levels of this adhesion molecule, and a CD56^dim^ population, expressing lower levels of this adhesion molecule, that represent about 90% of mature NK cells (16). Thus, phenotypically, human NK cells are defined as CD3^−^/CD56^+^/CD335 (NKp46)^+^ mononuclear cells that can be further divided into low density CD56^dim^ and high-density CD56^bright^ subsets (24). The NKp46 is a member of the highly conserved family of natural cytotoxicity receptor (NCR), a family of NK-activating receptors, also expressed by a small subset of cytotoxic T lymphocytes (25). The two NK cell subpopulations present specific localization, phenotype, and function. CD56^dim^ NK cells are the dominant subset in peripheral blood and are cytotoxic effector cells, which express high levels of FcγRIII (CD16), an immunoglobulin superfamily member, and CD57 (HNK-1, Leu-7). In addition, they present on their cell surface CXCR1 and the Chemerin receptor that play a role in NK cell recruitment into peripheral inflammatory sites. This subset, however, has a lower ability to produce cytokines in response to activation. On the other side, CD56^bright^ NK cells are present in secondary lymphoid organs and peripheral tissues. They express CCR7 that regulate their homing to lymphonodes, lack perforin, presenting little or no ability to spontaneously kill tumor cell targets, are CD16^−^, and mediate immune response by secreting pro-inflammatory cytokines, such as interferon-γ and tumor necrosis factor (TNF) alfa (26, 27).

The production of pro-inflammatory or immunosuppressive cytokines is another essential feature of NK cell, distinct from the secretion of cytotoxic granules, and NK cells use diverse activation signals to regulate these two functions in a differential way (16, 28).

As CD4+ T cells, also NK cells can be distinct into Natural killer type-1 (NK1), NK type-2 (NK2) (29), and NK regulatory cells (NKreg) (30). The different functional NK subsets are characterized by different expression of cell surface proteins and cytokines. NK1 cells are NK cells with activating signals, are mainly CD56^dim^ CD11b^+^ CD27^−^ NK cells, and produce IFN-g. NK2 cells are characterized by inhibitory signals, are mainly CD56^bright^ CD27^−^ CD11b^−^ NK cells, and produce type-2 cytokines, including IL-5 and IL-13; NKreg cells are mainly CD56^bright^CD27^+^ NK cells and play their immune regulatory effect by cytokines secretion or cell-to-cell contact (29, 31). The differentiation of NK cell subsets depends on the specific microenvironment in physiological or pathological conditions other than intrinsic regulation by various transcription factors.

In response to tumor ligands or intracellular pathogens, NK cells mainly produce Th1 type cytokines including IFN-g, TNF, and GMCSF which facilitate the activation of T cells, dendritic cells, macrophages, and neutrophils (32, 33). NK cells also produce cytokines with chemotactic action, including CCL3 (MIP-1α), CCL4 (MIP-1β), CCL5 (RANTES) which attract effector lymphocytes and myeloid cells toward inflamed tissues (34).

Similarly, different cells can produce inflammatory mediators that act on NK cells influencing their behavior. Dendritic cells play a pivotal role in this setting. These antigen-presenting cells, through the production of critical cytokines such as IL15, 12, 23, 27, and 18 (35–37), can enforce NK cell cytolytic activity. On the other hand, type 1 interferons, IL-12, IL-18, IL-27 released by dendritic cells are powerful activators of NK cell effector functions (38). IL2, produced by T cells, promotes the proliferation, cytotoxicity, and secretion of cytokines by NK cells (16). The NK cell functions can also be regulated by TGF beta released by regulatory T cells (Treg) (39, 40).

NK cells are key regulators of the immune response and of the cross-talk between innate and adaptive immunity, since they not only play a protective role from pathogen infections but also from excessive immune response to these agents (14). In fact, NK stimulation by various soluble factors (such as IL-15, type I IFN, IL-12, IL-18) can increase the maturation and activation of dendritic cells, macrophages, and T cells (41) but simultaneously NK can also present a cytotoxic action on immature dendritic cells, on activated T cells, and on hyper-reactive macrophages (42, 43).

All the described NK cell functions play an important role not only in physiological but also in pathological conditions, where NK cells can locally modulate several mechanisms of injury (16). Although many studies on human NK cells focused on peripheral blood, it is now clear that both CD56^dim^ and CD56^bright^ populate also healthy lymphoid and non-lymphoid organs including liver and kidneys. The kidney resident NK cells display a specific surface marker profile in different pathological conditions (44, 45). A significantly increased NK cell number is present in kidney biopsies from patients with chronic kidney disease (CKD). In this setting both NK CD56^dim^ and CD56^bright^ cells were increased in fibrotic renal tissue, but only CD56^bright^ correlated significantly with loss of kidney function (46), thus indicating that these cells, through the production of pro-inflammatory cytokines and IFN-g, can play an important role in fibrotic process and in the progression of kidney injury (46).

## NK Cells in Kidney Transplantation

NK cells in the transplanted kidneys are a heterogeneous population of innate lymphocytes with subset-specific functional roles and with complex functions during homeostatic and pathological conditions. In fact, their role in immune reactivity to solid-organ transplant is still controversial. It is well-known that NK cells might promote allograft injury. However, some evidences indicate that NK cells may play a significant role in the priming of allograft tolerance (47). Post-transplantation NK cell subsets can change also at the peripheral level when compared to pre-transplant cells, and these variations affect both the number and the phenotype (48), thus suggesting that NK cell immunoregulatory characteristics can largely influence the graft outcome.

### NK Cell Involvement in Acute and Chronic Allograft Rejection

Kidney graft rejection is classified pathologically into two types: T cell-mediated rejection (TCMR) and antibody-mediated rejection (ABMR) (49–51). Through various interactions with different cell types involved in the immunological response activated by organ transplantation, NK cells can contribute in different ways to the pathogenesis of both acute and chronic T cell-mediated and antibody-mediated rejection (47). Yagisawa et al. recently demonstrated, in a mice model of kidney transplantation, that acute kidney allograft rejection is induced by the presence of both NK cells and donor specific antibodies (DSA), whereas in the absence of NK cell activation the presence of DSA alone cannot induce acute antibody-mediated rejection, although it can still lead to late graft failure (52).

NK cells can also influence maturation of dendritic cells and the subsequent activation of T cells (53). Moreover, NK cells are an early source of IFN-g, which drive a Th1-type immune response. NK cells can interact directly with CD4+ T lymphocytes (54), increasing their reactivity, and these activities can induce acute rejection mechanisms.

**Figure 1:**
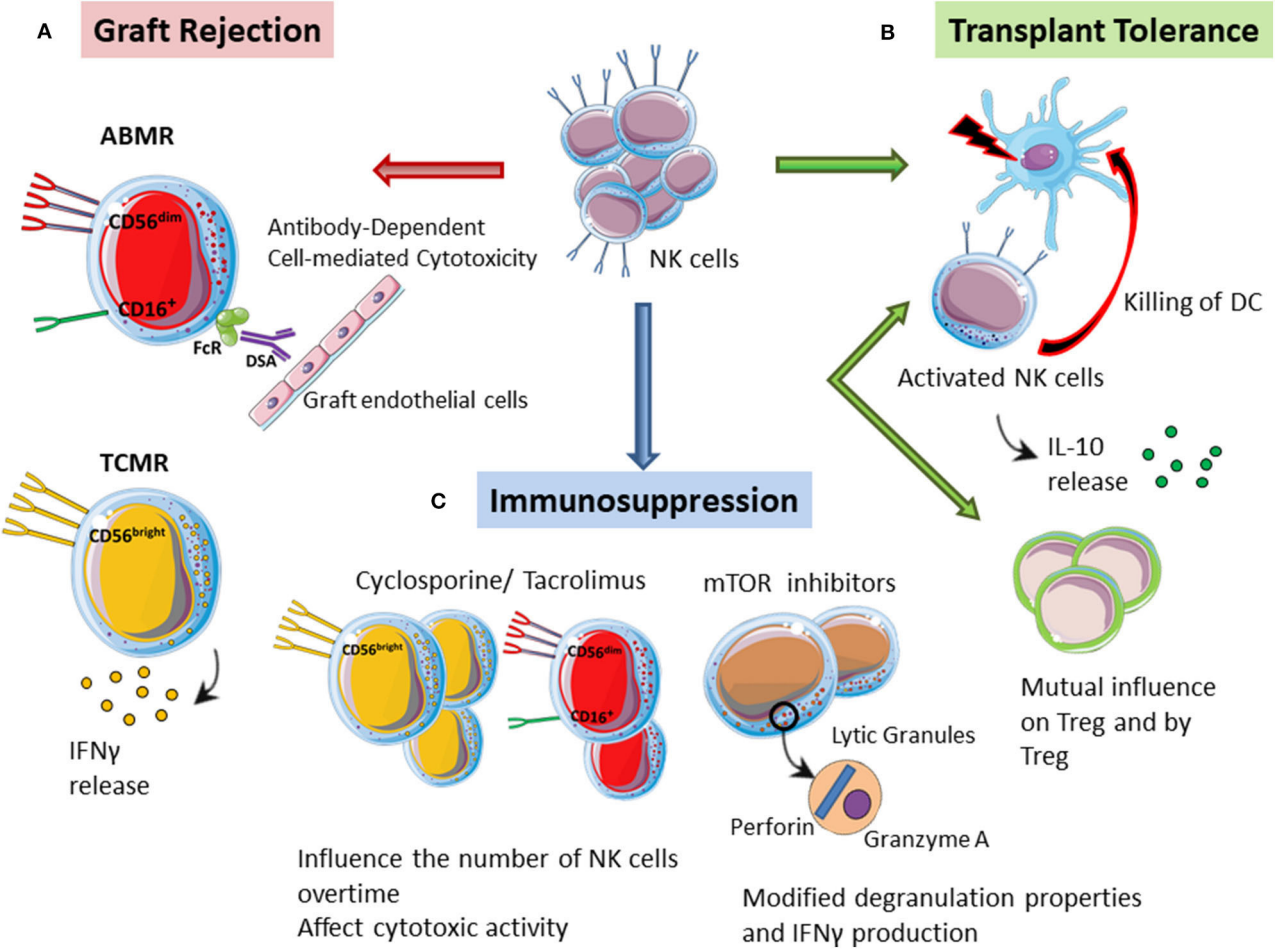
The multiple role of NK cells in kidney transplantation. **(A)** Graft rejection. CD56^dim^/CD16 NK cells can promote ADCC against the graft by interacting with DSA bound to the graft endothelial cells, thus driving antibody-mediated rejection (ABMR). CD56^bright^ NK cells can instead play a specific role in T cell-mediated rejection (TCMR) through the secretion of pro-inflammatory molecules such as IFN-g. **(B)** Transplant tolerance. Activated NK cells can directly kill donor-derived dendritic cells, thus promoting transplant tolerance. In mice tolerant models, NK cells can also produce high levels of IL10 thus showing tolerogenic ability. NK cells and Tregs might also influence each other by a mutual antagonism or by a temporary definition of their contribution in the induction of transplant tolerance. **(C)** Immunosuppression. Immunosuppressive drugs might modulate the phenotype of NK cells that can retain their ability to respond to stimulation. Moreover, immunosuppression can reduce the number of NK cells after kidney transplantation. Monitoring NK cell numbers and functions in transplanted patients under specific immunosuppressive regiments is important to control and predict the onset of infections and neoplasia.

Turner et al. (2) proposed a mechanism describing the pathogenic role of NK cells in human antibody-mediated rejection through the expression of CD16. This function can be triggered by anti-HLA antibodies, in particular by DSAs that represent a major risk factor for graft loss. NK cells have been identified in the peri-tubular capillaries of the biopsies of patients ABMR (55) where DSA bind the graft endothelial cells. Once bound to endothelial cells DSA can interact with FcγRIII present on NK cells inducing an ADCC against the graft (2). This model specifically involves the NK CD56^dim^ subset, expressing CD16, and recruited at the graft level. Thus, CD56^dim^/CD16 NK cells could represent the main NK subset involved in the pathogenesis of antibody-mediated rejection and responsible of ADCC on target cells into the graft (Figure 1A). Patients with DSA present, indeed, a reduced number of circulating CD56^dim^ NK cells compared to patients without anti-HLA antibodies or with non-DSA anti-HLA antibodies (56), and this observation might be the result of NK cytotoxic subset homing within the rejecting graft. Sablik et al. recently reported that there are no significant differences in the total percentage and distribution of NK cells, B cells, and T cells between patients with chronic active antibody-mediated rejection and control transplant recipients. However, antibody-mediated graft rejection is characterized by differences in the activation status of circulating monocytes, NK cells, and γδ T cells, mainly regarding the CD16 expression (57).

NK cells may play also a role in the pathogenesis of T cell-mediated rejection. Immunohistochemical characterization of graft infiltrating cells demonstrated that patients with acute T cell-mediated rejection are characterized by a higher number of CD56^+^ and CD57^+^ cells within the interstitial compartment, associated with interstitial inflammation and tubulitis, both characteristics of T cell-mediated rejection (58, 59). Authors also established a cut-off of 0.56 cells/mm^2^ both in the interstitial infiltrate and at the glomerular level, which was significantly associated with a worse graft survival (59). However, the main limits of these studies were the identification of NK cells based only on the expression of a single marker, CD56 or CD16, and the impossibility to identify the NK cell subpopulations involved in this context.

These findings suggest that NK cells need to be carefully evaluated, because variations in NK cell marker expression might be associated with the activation of different immune pathways in graft rejection. A recently published study, where authors isolated lymphomonocytes directly from graft biopsies and used a multi-color flow cytometry to define NK cell subsets involved in different graft rejection, confirmed this hypothesis (60). Biopsies from patients with T cell-mediated rejection showed an increased absolute number of CD56^bright^ NK cells while in the biopsies of patients with antibody-mediated rejection both CD56^bright^ and CD56^dim^ NK cells were increased. Only CD56^dim^ showed the expression of activation markers such as CD69 and high levels of cytotoxic effector molecules (perforin, granzyme A, and granulysin) in supernatants obtained from ABMR biopsies (60). Once again, these data highlight the importance of CD56^dim^ cells activation by the micro-environment featuring ABMR, where they can guide vascular damage. CD56^bright^ NK cells can instead play a specific role in TCMR through the secretion of pro-inflammatory molecules such as IFN-g (Figure 1A), which increase the recruitment of alloreactive T cells and up-regulate HLA alloantigens (MHC I and II) on graft target cells, making them more susceptible to cytotoxic killing (60).

Hidalgo et al. analyzed the transcriptional profiles of graft biopsies of patients with antibody-mediated rejection in the presence and absence of DSA (61). They demonstrated that the presence of DSAs was associated with 132 different transcripts, some in common with the T cell mediated rejection. By eliminating these shared transcripts, the authors identified 23 selective associated transcripts. Six of these 23 transcripts showed a high expression in NK cells, while the rest were mainly expressed at the endothelial level (61).

The different role of NK cells in T cell-and B cell-mediated rejections was also confirmed by the analysis of transcriptomic profiles of 403 kidney graft biopsies (62). Gene expression profiling of human kidney allografts identified high levels of NK cell transcripts in early T cell-mediated rejection, thus suggesting a distinct role for NK cells in this tubule-interstitial disease, while late biopsies showed increased number of NK cell transcripts in patients with antibody-mediated rejection, microvascular inflammation, and DSA. These data support the different role of NK cells in ABMR compared to the TCMR. Shin et al. also reported a positive correlation between the number of CD56^+^ cells and the severity of T cell-mediated rejection (63). In addition, it has been recently demonstrated that transcripts from activated NK cells are the only among those from leukocyte types that differentiate antibody- and T cell-mediated rejections and correlate with transplant outcome (55).

Several immune cells subtypes such as CD4^+^ and CD8^+^ T lymphocytes, monocytes/macrophages, dendritic cells, and NK cells infiltrate the kidney during graft rejection; however, kidney biopsies from ABMR patients are specifically characterized by a significant enrichment of NK cell transcripts, and activated NK cell infiltration can discriminate ABMR from TCMR and can predict graft failure after kidney transplantation (55). NK-cell depletion in mice models can, indeed, significantly attenuate the frequency and severity of antibody-mediated chronic rejection and the presence of NK cells is important in the pathogenesis of antibody-mediated graft lesions (64).

Although different cell types and different cell subsets are involved in ABMR and TCMR, respectively, NK cells and CD8 T cells, shared transcripts are expressed in the graft in both antibody- and T cell-mediated rejections, such as CD160, XCL1, TNFRSF9, and IFN-g, thus indicating possible similar effector systems, important in rejection mechanisms (65).

Our group has recently identified specific transcripts from NK cells also on peripheral blood mononuclear cells isolated from patients with antibody-mediated rejection compared to control transplant recipients with normal graft function and histology (Pontrelli P, personal communication). In particular, we observed that patients with antibody-mediated rejection are characterized by the increased presence of specific NK cell receptors that are essential for NK cell behavior. The key mediators of NK cell alloreactivity, in fact, are multiple receptors, KIRs, that predominantly recognize HLA Class I molecules (66). The different KIR expression on NK cells allows the ability of these cells to evaluate minute changes in MHC class I expression (67). Inhibitory KIRs expressed by NK cells of solid organ transplant recipients with donor mismatched for HLA KIR ligands may not recognize HLA class I molecules of donor and may dramatically induce NK cell alloreactivity against the graft (68, 69). Indeed, Littera et al. demonstrated, in a retrospective study including 174 donor/recipient pairs, a significantly higher risk of chronic rejection when recipient and donor pairs completely lacked the two KIR-HLA ligand combinations rKIR2DL1/dHLA-C2 and rKIR3DL1/dHLA-Bw4 corresponding to a low level of NK cell inhibition (70). Van Bergen et al. in a retrospective cohort study of 397 HLA-DR-compatible kidney transplantations demonstrated that KIR-ligand mismatching contributes to the rejection of human solid allografts as an independent risk factor in HLA-A-B-DR-compatible transplantations, indicating that suppression of NK-cell activity can improve the kidney graft survival (71).

NK (mis)matching between KIR receptors and HLA molecules can largely influence a transplant outcome and many studies confirmed the importance of NK KIR mismatching in graft-vs.-host disease (72). Recently, it has been demonstrated that in hematopoietic stem cell transplantation, activation of donor NK cells, in the absence of appropriate inhibitory ligands, can largely influence the outcome of transplantation. The specific analysis on KIRs genotype and HLA-A/B genotypes on a cohort of 100 patients with acute leukemia who received hematopoietic stem cell transplantation from their HLA-matched siblings suggested that an appropriate selection based on donor-recipient KIR genotypes and recipient HLA class I molecules can modulate the risk of host's disease and the efficacy of transplantation (73).

### How Can NK Cells Influence Transplant Tolerance?

Despite their essential role in allograft rejection, NK cells might also promote allograft tolerance (74). Immunological tolerance to a set of antigens is the absence of an immune response against those antigens, while normal responses to other antigens are preserved. Therefore, tolerance is an active antigen-specific process, is achieved under conditions that suppress the immune reaction, and is not just the absence of an immune response (75).

In specific settings, NK cells have potent immunoregulatory properties that promote tolerance induction. In a skin transplant model in mice it has been demonstrated that recipient's NK cells can contribute to the induction of graft tolerance by killing allogeneic antigen presenting cells (76). Donor antigen-presenting cells, in the absence of host NK cells, can survive and directly induce the activation of alloreactive T cells that are resistant to co-stimulatory blockade treatment (76). Thus, in those models in which NK cells have an altered function or are reduced, it will be difficult to obtain tolerance toward a MHC mismatched graft. After transplantation, in fact, both antigen presenting cells and T cells may represent potential targets of NK cell regulation (77). In this scenario, NK cells can be activated through different mechanisms: detection of the missing MHC I-self on the target cells, recognition of the Fc portion of the IgG, recognition of altered molecules on cells under stress conditions, inflammatory environment mediated by the cytokines produced by dendritic cells and T cells (78, 79). Activated NK cells can kill donor-derived dendritic cells through direct lysis (77), thus dampening the immune response and promoting a tolerogenic environment (Figure 1B). It is not yet clear which factors move NK cells toward immature donor-derived dendritic cells. Moreover, NK cells regulation could also affect recipient dendritic cells, thus influencing allograft antigen presentation (77). It is conceivable that NK cells might be able to integrate stimulating and inhibiting signals influenced also by T cell behavior, thus defining the final immune response (47).

The maintenance of transplant tolerance could be also associated with the production of IL-10 by NK cells (Figure 1B). Upon stimulation with glycolipids, such as galactosyl ceramide, NK cells produce high levels of IL-10 that can promote the development of regulatory dendritic cells (80). Moreover, NK cells from tolerant mice show high IL10 levels and can influence the immune response mediating heart transplant tolerance (81).

Lozano et al. described the presence of genes transcripts associated with NK cell-mediated cytotoxicity in the expression profiles of peripheral lymphocytes obtained from tolerant recipients, especially in liver transplant recipients (82). Even in kidney transplantation, the NK cell signature characterizes a tolerogenic action in recipients and kidney transplant patients with spontaneous operational tolerance are characterized by specific transcriptional profiles (82, 83). Dugast et al. showed that, although the frequency of circulating NK cells was normal in these spontaneously tolerant patients, these cells showed a reduced activation profile with a reduced expression of activating *KIR2DS5* gene, NKp46, and CD16 with a subsequent reduction in the effector functions of these cells including cytotoxicity and the release of cytokines such as IFN-g (84).

In the induction of tolerance by suppressing the immune response, Tregs play a leading role. Tregs are typically CD4^+^CD25^+^ and express the foxp3 transcription factor, which is the main inducer and regulator of Treg development and functions (85). CD4^+^CD25^+^T cells suppress the proliferation of CD4^+^ and CD8^+^ T lymphocytes. Thus, their major role is to shut down an immune reaction mediated by T cells and to suppress auto-reactive T lymphocytes that escaped the negative selection in the thymus (86). Tregs can influence the NK cell function in different ways, and this interaction can be positive in physiological conditions, such as pregnancy, or negative in some pathological conditions, such as autoimmune diseases or neoplasms, where Tregs suppress NK cells and inhibit their effector functions (87). On the other hand, NK cells maintain a complex crosstalk with different cells of the immune system (monocytes, B and T cells) (88–92) through direct contact or secretion of cytokines including TGF-beta. In correlation with higher TGF-beta level in inflammatory response, NK cells are able to induce Tregs (87, 93). However, how NK cells and Treg cells can influence each other in physiological and pathological conditions is still largely unknown.

A direct correlation between NK cells and Tregs in inducing tolerance is currently controversial (94). To date, most published evidences support the possibility of a mutual antagonism between NK cells and Tregs (94). An alternative proposal is that the reactivity of NK cells and Tregs are temporally distinct during the induction of tolerance (47). NK cells would induce tolerance in the first 3 weeks after transplantation by blocking dendritic cells and/or T cells that could start rejecting the graft, while Tregs, by maturing later, would maintain the long-term tolerance toward the graft (74). It is therefore possible that NK cells *per se* do not induce tolerance but simply allow the survival of the graft while the recipient develop a regulatory response (47) (Figure 1B).

## How Does Immunosuppression Influence NK Cell Behavior?

Information regarding the influence of immunosuppressive drugs on the activity of NK cells in transplant recipients is rather limited compared to T cells, which represent the main target of immunosuppressive therapies.

It has been demonstrated that certain KIR genotypes and their specific HLA class I ligands could affect kidney transplantation outcome by interfering with the efficacy of immunosuppressive drugs (70). The interference of KIR with therapy effectiveness has been already explored in allogenic transplantation of hematopoietic stem cells in chronic myeloid leukemia (95–97).

Immunosuppressive drugs might modulate the phenotype of NK cells after kidney transplantation, thus suggesting that NK cells can serve as sensors for immunosuppression and can be considered for personalized immunosuppression therapy adjustment (98). In fact, among kidney transplant recipients with a reduced expression of CD16 and CD56 on NK cells compared to healthy controls, patients in immunosuppressive therapy with tacrolimus showed more significant phenotypic changes on the expression of these markers than patients treated with cyclosporine or tacrolimus in combination with mTOR inhibitors (98). In addition, the presence of mTOR inhibitors *in vitro* also had functional consequences regarding de-granulation and IFN-g production (98) (Figure 1C). However, it is unclear whether these phenotypic changes of NK cells, induced by immunosuppressive drugs, may represent an activation signal of NK cells rather than functional exhaustion.

Hoffmann et al. demonstrated that NK cells of kidney transplant recipients under immunosuppression retain their ability to respond to stimulation since they produce equal amounts of IFN-g, perforin, and granzyme compared to NK cells from healthy individuals in response to strong, non-specific stimulation by PMA/Ionomycin (3). Thus, the inability of current immunosuppressive regimens to down-regulate the function of NK cells represents an opportunity from a therapeutic point of view, and new treatments targeted to activated NK cells and/or their effector functions should be explored.

However, immunosuppression may influence the number of NK cells over time. In patients treated with cyclosporine compared to patients treated with tacrolimus, the number of NK cells as well as the ratio CD56^dim^/CD56^bright^ is lower and the cytotoxic activity is reduced 1 year after transplantation (99). It will be useful in the future to routinely monitor and evaluate NK cell function in the context of specific algorithms to personalize immunosuppressive regimens (Figure 1C).

Monitoring NK cell number and especially NK cell function in transplanted patients is also important to control and predict the onset of infections and neoplasia (100). NK cells, indeed, play an important role in cancer defense, and the incidence of cancer is deeply increased after transplantation (101). Peraldi et al., in a cross-sectional multi-center case control study, demonstrated that kidney transplant recipients with cancer had a lower frequency of the cytokine-enriched CD56^bright^ NK cell subset compared to normal kidney graft recipients. The percentage of NKp46^+^NK cells in these patients was significantly reduced (45 vs. 53%, P = 0.001) along with a significant reduction in the ability of NK cells to degranulate CD107a+ cytolytic vesicles and to secrete IFN-g (102). In addition, Dendle et al. recently demonstrated that NK cell cytotoxic functions predict the appearance of severe infections in kidney graft recipients 2 years after transplantation better than NK number (103). Activated KIR genes have been associated with the protection from human Cytomegalovirus infection in renal transplantation (85), an infection associated with graft loss and reduced survival. In particular the presence after Cytomegalovirus infection of a specific subset of mature NK cells expressing the CD94/NKG2C-activating receptor can control the viral infection in kidney transplant recipients (104, 105).

## Concluding Remarks

The phenotype of NK cells in peripheral blood of kidney transplant recipients might be informative of the immune status after transplantation in terms of rejection vs. tolerance induced by immunosuppressive drugs.

A more careful evaluation of the number and function of these cells will allow us to balance the activation of mechanisms underlying graft rejection, favoring the immunological tolerance of the graft. This will achieve an equilibrium condition that allows the best survival of the graft and a reduction in the risk of developing malignancies or infections.

## Author Contributions

PP, GG, LG, and GS designed, wrote, and critically revised the review. FR and GC analyzed NK cell involvement in acute and chronic allograft rejection. All authors contributed to the article and approved the submitted version.

## Conflict of Interest

The authors declare that the research was conducted in the absence of any commercial or financial relationships that could be construed as a potential conflict of interest.
